# Steerable-filter based quantification of axonal populations at the developing optic chiasm reveal significant defects in *Slit2*^*−/−*^ as well as *Slit1*^*−/−*^*Slit2*^*−/−*^ embryos

**DOI:** 10.1186/1471-2202-14-9

**Published:** 2013-01-15

**Authors:** Matthew Down, David A Willshaw, Thomas Pratt, David J Price

**Affiliations:** 1 Institute for Adaptive and Neural Computation, University of Edinburgh, Edinburgh, UK; 2 Centre for Integrative Physiology, University of Edinburgh, Edinburgh, UK

**Keywords:** Axon guidance, Optic chiasm, Retinal axon, Slit, Steerable filter

## Abstract

**Background:**

Previous studies have suggested that the axon guidance proteins Slit1 and Slit2 co-operate to establish the optic chiasm in its correct position at the ventral diencephalic midline. This is based on the observation that, although both Slit1 and Slit2 are expressed around the ventral midline, mice defective in either gene alone exhibit few or no axon guidance defects at the optic chiasm whereas embryos lacking both Slit1 and Slit2 develop a large additional chiasm anterior to the chiasm’s normal position. Here we used steerable-filters to quantify key properties of the population of axons at the chiasm in wild-type, *Slit1*^*−/−*^, *Slit2*^*−/−*^ and *Slit1*^*−/−*^*Slit2*^*−/−*^ embryos.

**Results:**

We applied the steerable-filter algorithm successfully to images of embryonic retinal axons labelled from a single eye shortly after they have crossed the midline. We combined data from multiple embryos of the same genotype and made statistical comparisons of axonal distributions, orientations and curvatures between genotype groups. We compared data from the analysis of axons with data on the expression of *Slit1* and *Slit2.* The results showed a misorientation and a corresponding anterior shift in the position of many axons at the chiasm of both *Slit2*^*−/−*^ and *Slit1*^*−/−*^*Slit2*^*−/−*^ mutants. There were very few axon defects at the chiasm of *Slit1*^*−/−*^ mutants.

**Conclusions:**

We found defects of the chiasms of *Slit1*^*−/−*^*Slit2*^*−/−*^ and *Slit1*^*−/−*^ mutants similar to those reported previously. In addition, we discovered previously unreported defects resulting from loss of Slit2 alone. This indicates the value of a quantitative approach to complex pathway analysis and shows that Slit2 can act alone to control aspects of retinal axon routing across the ventral diencephalic midline.

## Background

The development of the complex connectivity of the nervous system involves the concomitant elongation and guidance of axons to specific targets. Growing axons are guided at their distal tips by growth cones, which move using relatively autonomous mechanisms to sense and respond to environmental cues. Some of these cues are molecules distributed in gradients that either attract specific growth cones up their concentration gradients (chemoattractants) or repel growth cones away from their sites of increasing concentrations (chemorepellents). One structure whose formation has provided an excellent model in which to study axon guidance is the optic chiasm of the mammalian visual system.

Retinal ganglion cell (RGC) axons from the two eyes converge on each other at the midline of the ventral part of the brain (ventral to the hypothalamus) where they either cross the midline (the contralateral tract) or turn away from it (the ipsilateral tract), forming the ×-shaped optic chiasm. The formation of this pathway is achieved through, the growth of RGC axons that exit the retina in tight bundles to reach the midline at a position ventral to the hypothalamus, where they either cross or do not cross depending on the retinal locations of their cell bodies. Guidance molecules that have been implicated in these regulatory tasks include Shh, which acts as a midline repellent to RGC axons except at the point where the chiasm forms [1], EphrinB2 and its receptor EphB1, which are critical for ipsilateral/contralateral sorting [2], and the extracellular secreted molecules Slit1 and Slit2, which signal via Robo receptors to constrain axons to the region of the chiasm [3-6].

An interesting aspect of Slit1 and Slit2 function at the chiasm is that, although both are expressed at the hypothalamic ventral midline and RGC growth cones respond to individual Slits [7-9], mice defective in either gene alone have been described as exhibiting either few or no RGC axon guidance defects at the optic chiasm [10]. By contrast, in double-mutant mice lacking both Slit1 and Slit2, a large additional chiasm develops anterior to the chiasm’s normal position [10]. These results indicate that Slit proteins co-operate to establish a corridor through which the axons are channelled, thereby helping define the site in the ventral diencephalon where the optic chiasm forms.

The nature of the co-operation between Slit1 and Slit2 at the optic chiasm, for example whether one plays a stronger a role than the other, is not clear. One of the main reasons is that previous analyses of the structure of the optic chiasm and the routes taken by the axons it contains rely on non-quantitative anatomical descriptions of its appearances after most or all of its axons have been labelled. The complexity of the chiasm and the large number of axons that it contains make it difficult to carry out quantifications that might reveal effects of the Slits individually. In this work we adapted a method based on second-derivative Gaussian steerable filters [11] to allow us to obtain quantitative information on the positions, curvatures and orientations of developing axons in large tracts such as the optic chiasm. Steerable filters have been used successfully to quantify key properties of neuronal processes *in vitro*[12], where the separation of the processes makes them clearer and easier to analyze than *in vivo*. Using a steerable-filters-based technique tailored specifically for our purposes, we found previously unreported defects at the optic chiasm of *Slit2*^*−/−*^ single-mutant mice that correlated with the normal expression pattern of Slit2. Our results suggest that Slit2 makes a greater contribution than Slit1 to the guidance of RGC axons at the optic chiasm. The application of a similar approach to the analysis of other mutants and similarly complex pathways might be a profitable way of finding hitherto undetected defects in some strains.

## Results

### Quantitative analysis of labelled axons at the wild-type chiasm

**Figure 1:**
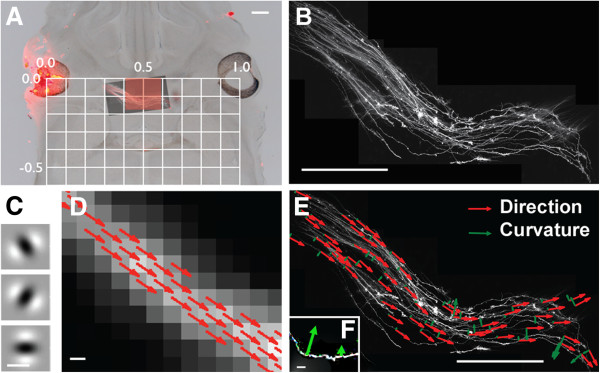
**Applying steerable filters to a DiI image of the developing mouse optic chiasm.** (**A**) A 150 μm-thick horizontal section of an E13.5 mouse brain into whose left eye DiI had been injected. An image of the chiasm taken with fluorescence was superimposed, revealing the DiI labelled axons. A grid whose baseline ran between the centers of each eye was used to align images from multiple embryos. The red shaded rectangle highlights the region that was selected for statistical comparison in sets of mice of different genotypes. Scale bar = 200 μm. (**B**) Magnification of the DiI labelled chiasm from A. Scale bar = 200 μm. (**C**) 2nd-derivative Gaussian filters of different orientations used to analyze images: among these examples, the top filter would respond best along the axon in D. Scale bar = 1 μm. (**D**) High magnification of a segment of axon from B. Arrows, which are associated with each pixel along the centre of the axon and point away from the injected eye, are aligned with the filter orientations that gave the best responses. Scale bar = 2 μm. (**E**) The result of applying the full algorithm to the image in B: red arrows represent direction, green arrows represent curvature. Only a small subset of arrows has been plotted, for clarity. Scale bar = 200 μm. (**F**) Examples of two arrows at different positions on a single axon (surrounding axons removed for clarity) showing how vector length is proportional to curvature, with the direction of the arrow indicating the direction of curvature. Scale bar = 10 μm.

We first examined DiI-labelled axons at the optic chiasm of wild-type mice (Figure 1). Figure 1A shows an example of the optic chiasm in a horizontal section at embryonic day 13.5 (E13.5). E13.5 was selected because retinal axons have recently crossed the midline at this age, having first penetrated the diencephalon at E12.5 Erskine et al., [8]. It is, therefore, the earliest age offering the opportunity to observe potential defects of guidance at the chiasm in mutants.

Figure 1B is a high magnification view of the same chiasm as in Figure 1A, showing detail of the DiI labelled axons. Axons such as these were analyzed using Gaussian steerable filters, examples of which are illustrated as oriented spatial functions in Figure 1C. The filters were convolved with the images of DiI-labelled chiasms. Only data derived from points along axons were retained; points that were not on axons were excluded using non- maximum suppression, which removed data from areas that did not lie on ridges of high intensity with respect to the surrounding landscape of the image. Figure 1D illustrates the outcome: the orientations of filter that gave the best response at each position along the axon are shown as arrows pointing away from the DiI-injected eye (referred to as directions). An example of applying the algorithm to all the axons at the chiasm is illustrated in Figure 1E, which shows only a small subset of directions (or oriented vectors) for purposes of clarity. The subset is <0.01 of the size of the full set, allowing the arrows to be shown enlarged compared to those in Figure 1D. The algorithm provides a method to quantify automatically a vector field representing the orientations of axons in a given image and also the curls of the vector fields, which measure the degree of curvature of axons at each point. Notice that the arrows representing direction (red arrows in Figure 1D,E) have a constant magnitude whereas those representing curvature (green arrows in Figure 1E,F) have both direction and length, with the length proportional to the amount of turning.

### Axon locations in Slit1^−/−^, Slit2^−/−^ and Slit1^−/−^; Slit2^−/−^ embryos

**Figure 2:**
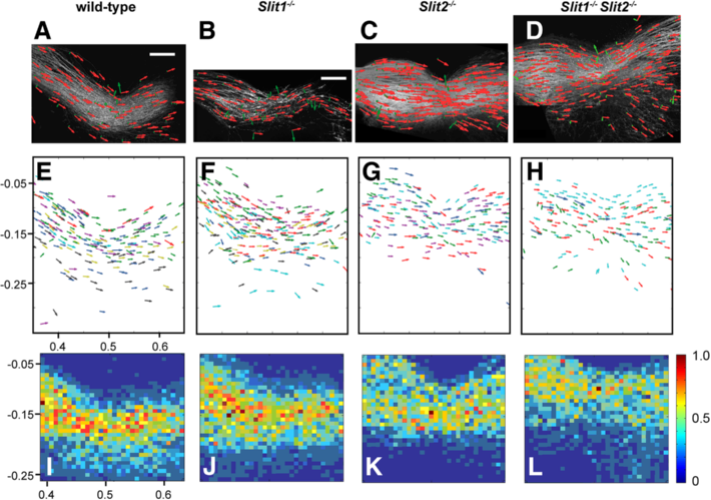
**Combining data from multiple embryos with different *Slit1 *and *Slit2 *genotypes.** (**A-D**) Images depict examples of chiasms analyzed using the steerable filter algorithm, with conventions as in Figure 1E. Scale bars = 200 μm. (**E-H)** Plots show the direction vector fields overlaid (using the grid system in Figure 1A) from all embryos of each genotype, with each colour representing data from a different embryo. Only small subsets of arrows from each embryo have been plotted, for clarity. (**I-L**) Heat-maps: In each square we calculated the mean number of arrows, including all arrows from all embryos of each genotype (these values for mean densities of arrows are proportional to the mean densities of axon). A colour scale is used to illustrate the spatial distribution of mean densities on a relative scale for each genotype. A, E, I, wild-type, n = 9; B, F, J, *Slit1*^−/−^, n = 11; C,G,K, *Slit2*^−/−^, n = 5; D,H,L, *Slit1*^−/−^*Slit2*^−/−^, n = 4.

Figure 2 illustrates the results of applying the steerable filter algorithm to sets of E13.5 embryos that were wild-type (Figure 2A,E,I; n = 9 embryos), *Slit1*^−/−^ (Figure 2B,F,J; n = 11 embryos), *Slit2*^−/−^ (Figure 2C,G,K; n = 5 embryos) or *Slit1*^−/−^*Slit2*^−/−^ (Figure 2D,H,L; n = 4 embryos). Figure 2A-D shows individual examples of chiasms analyzed as outlined in Figure 1. As can be seen, there is considerable variation in the appearance of these examples, particularly between those that are *Slit2*^−/−^ or *Slit1*^−/−^*Slit2*^−/−^ and those that are wild-type or *Slit1*^*−/−*^. To assess whether this variation can be accounted for by differences in genotype, we combined results from applying the algorithm to all embryos of each genotype. Figure 2E-H shows examples of vectors (i.e. subsets, as described above) representing axonal directions from all embryos of each genotype. Data from different embryos are shown in different colours, providing a sample of the routes taken by axons in all embryos of each genotype. Data from embryos within each genotype group were aligned using the grid system shown in Figure 1A. The mean distance between the centre of the two eyes did not vary significantly with genotype, varying by < 8% between groups. The plots in Figure 2E-H suggest an anterior shift in the position of the optic chiasm in *Slit2*^−/−^ and *Slit1*^−/−^*Slit2*^−/−^ embryos compared to wild-type and *Slit1*^*−/−*^ embryos.

To test this statistically, the area of each chiasm was split into a 32 × 32 grid. Figure 2I-L shows the spatial distributions of the mean numbers of oriented vector fields (i.e. the total numbers of arrows, as exemplified in red in Figure 1D) across the chiasms of all embryos of each genotypes (higher numbers are towards the red end of the spectrum). All vectors were included (i.e. not the samples explained above and used for illustrative purposes in Figures 1 and 2). These values were proportional to the mean densities of axon within each square for each genotype, since in all cases analysis with steerable filters was done at constant intervals and filtering using non-maximum suppression prevented the inclusion of data from areas that contained no axons. Note that while these values are proportional to the densities of axon within each square, and can therefore be used to examine distributions of axon, they can not be used to derive values for the absolute numbers of individual axons across the chiasm. Our approach does not attempt to trace individual axons and can not, therefore, give their absolute numbers.

**Figure 3:**
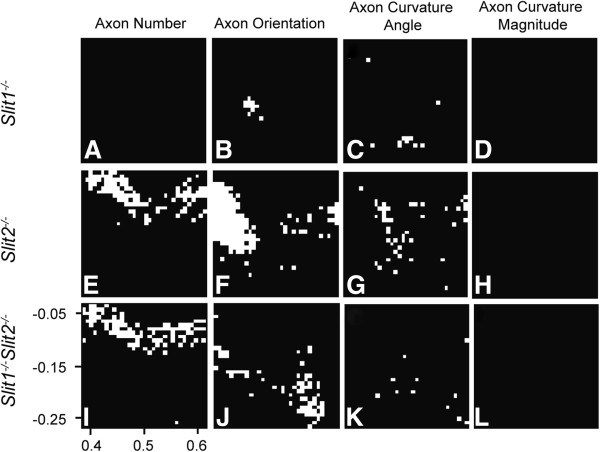
**Areas of statistically significant differences between wild-type and mutant chiasms.** In each panel the white squares indicate positions that differ significantly from wild-type; Student’s *t*-test was applied for axon number and Watson-Williams test for orientation and curvature. All tests were controlled for false positives [14]; α = 0.05; see Materials and Methods). Columns show differences in mean axon number, axon orientation, angle of axon curvature and magnitude of axon curvature per grid square. (**A-D**) *Slit1*^*−/−*^ embryos; (**E-H**) *Slit2*^*−/−*^ embryos; (**I-L**) *Slit1*^*−/−*^*Slit2*^*−/−*^ embryos. Wild-type, n = 9; *Slit1*^−/−^, n = 11; *Slit2*^−/−^, n = 5; *Slit1*^−/−^*Slit2*^−/−^, n = 4.

The graphs in Figure 2I-L suggest an anteriorization of the population of chiasmatic axons in *Slit2*^−/−^ and *Slit1*^−/−^*Slit2*^−/−^ embryos. For each area of the chiasm we tested for significant differences (taking account of multiple testing, see Methods) between the values from wild-type and *Slit1*^−/−^, *Slit2*^−/−^ or *Slit1*^−/−^*Slit2*^−/−^ embryos: results are plotted in Figure 3A,E, I. There were no significant differences between wild-type and *Slit1*^−/−^ embryos (Figure 3A), but significantly larger densities of axon were located in abnormally anterior positions in both *Slit2*^−/−^ and *Slit1*^−/−^*Slit2*^−/−^ embryos (Figure 3E,I). There were no significant differences between *Slit2*^−/−^ and *Slit1*^−/−^*Slit2*^−/−^ embryos (not shown).

### Axon orientations and curvatures in Slit1^−/−^, Slit2^−/−^ and Slit1^−/−^; Slit2^−/−^ embryos

The same procedure was then applied to values of orientation, curvature angle and curvature magnitude from each area across the chiasm. These values were the averages of the vectors within each square of the 32 × 32 grid and, therefore, took no account of the trajectories of *individual* axons, e.g. whether they crossed each other or not. Comparison of wild-type and *Slit1*^−/−^ embryos showed only a few areas returning statistically significant differences in either axon orientation or angle of curvature (Figure 3B,C). Comparison of wild-type and either *Slit2*^−/−^ or *Slit1*^−/−^*Slit2*^−/−^ embryos showed many more areas returning statistically significant differences in axon orientation, many of which were located lateral to the midline (Figure 3F,J; the midline runs vertically through the centre of each panel). In *Slit1*^−/−^*Slit2*^−/−^ double-mutant embryos there were also significant abnormalities of axonal orientations in a posterior area contralateral to the injected eye (areas in the bottom right of Figure 3J) that were not present in *Slit2*^−/−^ mutants. Regarding the angle of axon curvature, most differences between genotypes were found around the midline (Figure 3G,K). No comparison returned any significant differences in magnitudes of curvature (Figure 3D,H,L). Overall, these data indicate that, in *Slit2*^−/−^ and *Slit1*^−/−^*Slit2*^−/−^ embryos, many axons are oriented abnormally in their route across the chiasm, an observation that agrees with our finding described above that many axons are mislocated. In *Slit1*^−/−^ embryos there were few axon orientation defects, in line with there being no detectable axonal mislocation.

### Comparison of axonal defects with patterns of Slit expression

**Figure 4:**
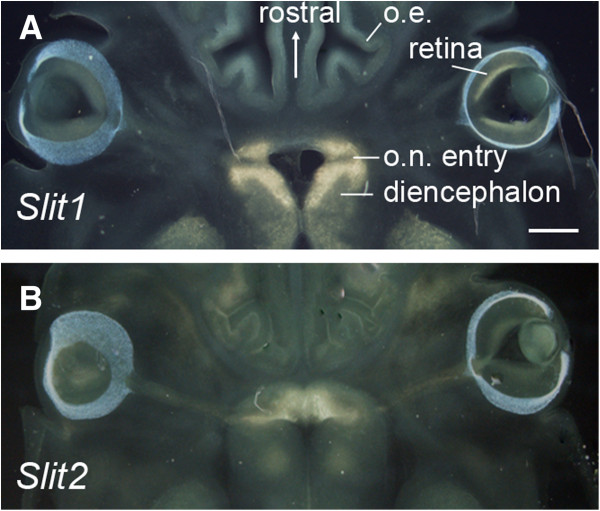
**Expression of Slit1 and Slit2 around the E13.5 optic chiasm.** In situ hybridizations on horizontal sections through the ventral diencephalon: areas of expression appear pale yellow. Expression of *Slit1* is strong around the point of entry of the optic nerve (o.n.); expression of *Slit2* is strong anterior to this point. Olfactory epithelium = o.e. Scale bar = 200 μm.

In agreement with previous studies, in situ hybridizations at E13.5 revealed strong *Slit1* mRNA expression both anterior and posterior to the junction of the optic nerve and the brain (Figure 4A) [8] whereas expression of *Slit2* was strongest anterior to the point of entry of retinal axons (Figure 4B) [7,8]. The analysis above revealed that many retinal axons of *Slit2*^−/−^ and *Slit1*^−/−^*Slit2*^−/−^ mutants crossed at abnormally anterior locations and here we examined the spatial relationship between the orientations of these axons and the normal *Slit2* expression pattern.

**Figure 5:**
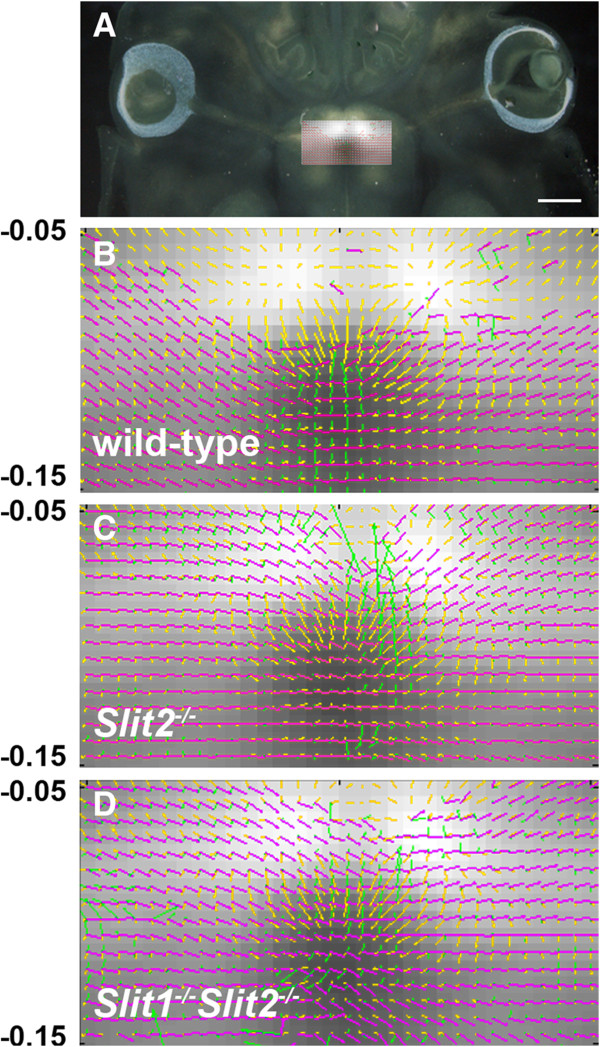
**Axonal defects in *Slit2***^***−/−***^**and *Slit1***^***−/−***^***Slit2***^***−/− ***^**embryos occur where Slit2 is normally expressed.** (**A**) The area of axonal analysis is superimposed on an in situ hybridization for *Slit2*. Scale bar = 200 μm. (**B-D**) Mean vector orientation (red/magenta), direction and magnitude of curvature (green) and magnitude and direction of *Slit2* gradient (yellow) are shown for each grid square for each genotype shown. Wild-type, n = 9; *Slit2*^−/−^, n = 5; *Slit1*^−/−^*Slit2*^−/−^, n = 4. Data are superimposed on an image showing the average intensities within each grid square of *Slit2* expression in wild-types (n = 3 in situ hybridizations).

Using the same strategy described above for analysis of axons, we combined data from three separate comparably-developed in situ hybridizations to give a map of the average staining intensity for *Slit2* across the ventral midline in 150 μm horizontal sections at the level of retinal axonal entry. The system illustrated in Figure 1A was used to align data on gene expression in wild-types with data on retinal axons from wild-type or mutant embryos. Results are shown in Figure 5. The following vector-fields were obtained. For axons, mean orientations (red/magenta in Figure 5B-D) and mean directions and magnitudes of curvature (green in Figure 5B-D) were obtained within each square in the 32 × 32 grid (described above), using data from all embryos of each genotype. Also within each square, the mean vector representing the gradient of *Slit2* expression was calculated (yellow in Figure 5B-D; the lengths of the lines represent the magnitudes of the gradient with arrowheads pointing from high to low intensity of label). The location of these vector fields in the brain is shown in Figure 5A.

Axons were labelled from the left eye. In wild-types (Figure 5B) labelled axons about 200μm to the left of the midline were oriented roughly 45–75° relative to the midline. They turned to run roughly orthogonal to the midline as they as they approached it (note the green vectors representing curvature concentrated near to the midline in Figure 5B). In taking this course, the axons were oriented roughly (± about 30°) orthogonal to the vectors representing the *Slit2* gradient and, therefore, avoided the anterior region of high *Slit2* expression (Figure 5B). In contrast, axons on the left of the midline in *Slit2*^*−/−*^ mutants were oriented roughly orthogonal to the midline throughout their approach, thereby entering the anterior areas where *Slit2* would normally be expressed. Many turned at the midline to exit through the contralateral area of high *Slit2* expression (Figure 5C). A similar pattern was observed in *Slit1*^−/−^*Slit2*^−/−^ mutants (Figure 5D).

All these analyses together provide a consistent picture in which loss of either Slit2 alone or Slit1 and Slit2 together result in many retinal axons being misoriented on approach and exit from the ventral midline and many being located abnormally anteriorly, where Slit2 would normally be expressed. It appears that Slit2 is required to prevent retinal axons from taking this anterior route.

## Discussion

In this study we have shown it is possible to apply relatively simple image analysis methods to static images of developing axons, quantify key properties relevant to the study of axon guidance and combine data from multiple embryos to make statistical comparisons between groups sharing a particular property (here their genotype). Since it is very difficult to trace individual axons in dense axonal tracts *in vivo*, our approach was to determine the average positions, directions and curvatures of populations of axons. Data were combined from multiple embryos so as to allow statistical comparisons that took into account variations between individual embryos or introduced by technical factors such as the degree of fixation, the amount of tracer injected or the precise plane of section in each individual. Using this approach, we detected the previously-described defect of chiasm development in *Slit1*^−/−^*Slit2*^−/−^ mutants [10]. The real value of additional objectivity was demonstrated by our detection of hitherto unreported defects in *Slit2*^−/−^ mutants.

Based on their qualitative examination of mutant chiasms, Plump et al. [10] stated that they were unable to detect defects at the chiasm of *Slit2*^−/−^ mutants, with the caveat that they could not exclude defects that were beyond the sensitivity of their experiments. In fact, in the example that they show of labelled axons at the chiasm of *Slit2*^−/−^ mutants (Figure 3 of their paper) the anteroposterior width of the tract at the midline is about double the width of the wild-type. Our quantitative results indicate that there is indeed a defect of the *Slit2*^−/−^ chiasm with many axons mislocated abnormally anteriorly. Our results agree with the conclusion of Plump et al. [10] that there are no major defects at the chiasm of *Slit1*^−/−^ mutants. Regarding the comparison of *Slit2*^−/−^ and *Slit1*^−/−^*Slit2*^−/−^ embryos, we found significant misorientation of some of the posterior axons contralateral to the injected eye in double mutants, but otherwise they were similar to single mutants. Such misorientation of posterior axons was not found in *Slit1*^−/−^ mutants, indicating that there are abnormalities of double mutants that are not present in either mutant alone. Overall, however, our results indicate that, early in the formation of the chiasm, Slit2 plays a more powerful role than Slit1 in constraining the growth of axons to their correct location across the ventral midline.

While our results suggest a greater similarity between the effects of loss of Slit2 alone and loss of both Slit1 and Slit2 than was suggested by Plump et al. [10], the results of the two studies are in fact not strictly comparable. Plump et al. [10] reported a major difference between the *Slit2*^−/−^ and *Slit1*^−/−^*Slit2*^−/−^ genotypes at E15.5, which is two days later than our findings. Although they reported defects of the double mutants at earlier ages, including E13.5, that look very similar to those found here, they did not provide data on *Slit2*^*−/−*^ embryos at earlier ages. This raises the interesting possibility that the effects of losing both Slit1 and Slit2 become progressively more severe than those of losing Slit2 alone as the chiasm develops from E13.5 to E15.5.

The *Slit*^*−/−*^ phenotypes reported here have a striking correlation to the ectopic projection of *Slit*^*−/−*^ retinal axons to the contralateral eye that we reported previously. In wild-type and *Slit1*^*−/−*^ embryos the inter-retinal projection is relatively small indicating that Slit1 is dispensable for keeping retinal axons out of the opposite eye. In contrast, loss of *Slit2* function results in a dramatic increase in the size of the inter-retinal misprojection and a further increase occurs in *Slit1*^*−/−*^*; Slit2*^*−/−*^ embryos [6]. It is easy to imagine that these phenotypes are causally linked: those retinal axons that cross the midline in aberrant positions in *Slit* mutants are liable to end up on a track which predisposes them to misproject to the opposite eye. This provides an example of how the novel analysis techniques described here can shed extra light on axon guidance phenotypes previously observed using more traditional techniques.

The effect of Slit2 is likely explained by its spatial pattern of expression, which was studied here by in situ hybridization due to the lack of suitable antibodies. Clearly, it would be preferable to examine the distribution of Slit proteins, and our analysis of this issue is based on the assumption that the protein distribution would approximate to the mRNA distribution at a tissue level. *Slit2* mRNA is expressed in the ventral midline in a position anterior to the normal chiasm. It is straightforward to understand how loss of this expression might be a critical factor allowing axons to cross in an abnormally anterior position. The effects of Slit1 loss are intriguing and less easily explained. Slit1 is normally expressed both anterior and posterior to the point of entry of retinal axons. Its loss in combination with that of Slit2 causes some misorientation of posterior axons after they have crossed the midline, but why this defect is not detected in single *Slit1*^*−/−*^ embryos is not clear. It appears that the presence of other factors provides sufficient guidance even in the absence of Slit1 and that Slit2 might be one of these other factors. How Slit1 and Slit2 cooperate to prevent contralateral posterior misguidance is not clear. It is possible that Slit2 prevents axons approaching the midline from acquiring abnormalities that predispose some of them to require Slit1 repulsion from posterior contralateral territory. In this scenario Slit1 repulsion would only be required if Slit2 is lost.

## Conclusion

Our results identify a previously undetected but important role of Slit2 alone at the chiasm, while also showing some degree of cooperation between Slit1 and Slit2 and a lack of an obvious role for Slit1 alone, as suggested by previous work (Plump et al., 2002). We suggest that the approach adopted here can increase the sensitivity for detecting axonal defects in mutant strains and might be adopted more widely in the future.

## Methods

### Mice and mutant alleles

The licence authorising this work was approved by the University of Edinburgh Ethical Review Committee of 22^nd^ September 2008 (application number PL35-08) and by the Home Office on 6^th^ November 2008 (licence number 60/3913). Animal husbandry was in accordance with the UK Animals (Scientific Procedures) Act 1986 regulations. *Slit1* and *Slit2* mutant alleles were described by Plump et al. [10]. The *Slit1*^*-*^ allele was identified by multiplex PCR using primers 5-ACCCTTAGCTTCTACCAACC-3, 5- TCTCCTTTGATCTGAGACCG-3 and 5-AGGTTTCTCGAGCGTCATAG-3: the wild-type allele gives a 544bp product, the mutant allele gives a 393bp product. The *Slit2*^*-*^ allele was identified by multiplex PCR using primers 5-AAGACCTGTCGCTTCTGTCAG- 3, 5-AAACAGGTTTCTACCGCACG-3, and 5-AAGTCTAGTAGAGTCGAGCG-3: the wild-type allele gives a 600bp product, the mutant allele gives a 350bp product. All mice were C57BL6.

### DiI tract tracing

E13.5 embryonic heads were fixed at 4°C in 4% paraformaldehyde in phosphate buffered saline (PBS) overnight, and 1,1-dioctadecyl-3,3,3,3-tetramethyl-indocarbocyanine perchlorate (DiI) crystals (Invitrogen, San Diego, CA) were placed into the optic cup of one eye, after removal of the lens, to label axons leaving the retina. Heads were returned to 4%paraformaldehyde in the dark at room temperature for 4 weeks to allow tracers to diffuse along axons. Heads were then sectioned (150 μm) with a vibratome, cleared in 9:1 glycerol: PBS and mounted in Vectashield (Vector Laboratories, Burlingame, CA). Images were acquired at ×40 using a Zeiss Axiovert confocal LSM 510 microscope in tile-scan mode to collect serial optical sections, which were then combined to create a projection. The section thickness was sufficient to include the entire chiasm but where, due to the position of the cuts, part of it was in a second section, both sections were imaged and a composite was generated so that the entire depth of the chiasm was analyzed in all cases.

### Image analysis

Images were imported into MATLAB (MathWorks, Natick, MA) and convolved with a bank of three 2D 2nd-derivative Gaussian filters of width 1.8 μm. The maximum filter response was calculated. The method is described in full in Freeman and Adelson [11]. Imaging was done with consistent confocal settings chosen to avoid saturation at high magnification. Rather than adjust settings, where small areas of saturation did occur and the filters did not activate, vector orientations in the saturated area were interpolated based on the orientations the vectors were taking on the edges of that area. In practice, this approach only applied in very small areas in some images. Non-maximum suppression was then performed by considering the two orthogonal pixels at any given point in the direction vector field and keeping it if both these flanking pixels gave a lower filter response. A global threshold was then applied that removed all pixels for which the intensity was less than twice the median intensity across all pixels or whose filter response was less than twice the median. The orientation vector field was then smoothed with a Gaussian of 30 μm to remove noise (which is of a higher frequency) and the curl of this vector field was calculated to give the curvature of the axon trajectories at any given point. Non-maximum suppression was applied a second time.

### Statistical comparison of genotypes

To compare different genotypes with respect to axon number, axon direction and axon curvature across the image, the vector fields were rotated and scaled to a universal grid with the left eye of the embryo defining the origin and the right eye defining the position (0, 1), as shown in Figure 1A. The area of the chiasm was split into a 32 × 32 grid covering the region (0.4 : 0.6, 0.05 : 0.25), highlighted in Figure 1A. The axon number, mean axon orientation, mean curvature direction and mean curvature magnitude were put into each of these bins for every sample. A Student’s *t*-test was applied for axon number and the Watson-Williams test was used to compare axon orientations and angle of curvature between samples, as provided by the CircStat MATLAB toolbox (circular statistics is appropriate for analysis of curvatures and orientations) [13]. To control for false positives, FDR (False Discovery Rate) [14] was used, given the large number of hypotheses present. For all tests we took α = 0.05. These methods are commonly used for dealing with multiple comparisons in other areas of spatial statistics.

### In situ *hybridization*

Embryonic heads were fixed overnight in 4% paraformaldehyde in phosphate buffered saline (pH 9.5 at 4°C). In situ hybridizations for Slit1 or Slit2 used 100 μm vibratome sections and digoxigenin-labeled antisense riboprobes synthesed from rat cDNAs encoding *Slit1* and *Slit2* as templates, as previously described Erskine et al., [8].

## Competing interests

The authors declare that they have no competing interests.

## Authors’ contributions

MD carried out the experimental work and the image analysis, contributed to experimental design and selected the method of analysis. DW, TP and DP supervised the work, contributed to experimental design and selection of methodology. MD, TP and DP wrote the manuscript. All authors read and approved the final manuscript.
